# Molecular Mechanisms of Trastuzumab-Based Treatment in HER2-Overexpressing Breast Cancer

**DOI:** 10.5402/2012/428062

**Published:** 2012-11-22

**Authors:** Rita Nahta

**Affiliations:** ^1^Department of Pharmacology, School of Medicine Emory University, Suite 5001, 1510 Clifton Road, Atlanta, GA 30322, USA; ^2^Department of Hematology and Medical Oncology, School of Medicine Emory University, Atlanta, GA 30322, USA; ^3^Winship Cancer Institute, Emory University, Atlanta, GA 30322, USA; ^4^Molecular and Systems Pharmacology Program, Graduate Division of Biological and Biomedical Sciences, Emory University, Atlanta, GA 30322, USA

## Abstract

The past decade of research into HER2-overexpressing breast cancer has provided significant insight into the mechanisms by which HER2 signaling drives tumor progression, as well as potential mechanisms by which cancer cells escape the anticancer activity of HER2-targeted therapy. Many of these preclinical findings have been translated into clinical development, resulting in novel combinations of HER2-targeted therapies and combinations of trastuzumab plus inhibitors of resistance pathways. In this paper, we will discuss proposed mechanisms of trastuzumab resistance, including epitope masking, cross signaling from other cell surface receptors, hyperactive downstream signaling, and failure to induce antibody-dependent cellular cytotoxicity. In addition, we will discuss the molecular mechanisms of action of dual HER2 inhibition, specifically the combination of trastuzumab plus lapatinib or trastuzumab with pertuzumab. We will also discuss data supporting therapeutic combinations of trastuzumab with agents targeted against molecules implicated in trastuzumab resistance. The roles of insulin-like growth factor-I receptor and the estrogen receptor are discussed in the context of resistance to HER2-targeted therapies. Finally, we will examine the major issues that need to be addressed in order to translate these combinations from the bench to the clinic, including the need to establish relevant biomarkers to select for those patients who are most likely to benefit from a particular drug combination.

## 1. Introduction

The *HER2* (*erbB2*/*neu*) receptor tyrosine kinase gene is amplified and overexpressed at the protein level in 20–30% of metastatic breast cancers.* HER2* overexpression represents an example of oncogene addiction in many of these cancers, as HER2 blockade or kinase inhibition achieves durable responses in many patients with metastatic HER2-overexpressing breast cancer. The first-line treatment for this subtype of breast cancers is the HER2 monoclonal antibody trastuzumab. In combination with cytotoxic chemotherapy, trastuzumab has revolutionized treatment and clinical outcome for patients whose breast tumors express high levels of the HER2 protein. Despite remarkable success, response rates are usually limited in duration, suggesting that the development of resistance is a clinical problem. Research published during the past decade has identified multiple molecular mechanisms contributing to trastuzumab resistance. In addition, recent studies have suggested novel combinations of drugs that will benefit patients who have shown disease progression on prior trastuzumab treatment, including combinations of HER2-directed drugs and drugs targeted against the molecular drivers of resistance. In this paper, we will discuss potential mechanisms of resistance to trastuzumab, and mechanisms of action of dual HER2 inhibition. We will also discuss data supporting therapeutic combinations of trastuzumab with agents targeted against molecules implicated in trastuzumab resistance. Finally, we will examine the major issues that need to be addressed in order to translate these combinations from the bench to the clinic. 

## 2. Discovery of the HER2 (erbB2/neu) Oncogene

 In 1982, a seminal paper was published showing that DNA from NIH/3T3 cells transformed with genetic material from chemically-induced rat neuroblastomas could ultimately result in development of fibrosarcomas when injected into mice [1]. Sera from these mice immunoprecipitated a 185-kiloDalton phosphoprotein from cells transfected with the neuroblastoma-derived DNA [1]. This phosphoprotein was found to be related to the epidermal growth factor receptor (EGFR) and was called *neu* [2]. The transforming version of *neu* (*neuT*) and the wild-type gene were subsequently cloned, and *neuT* was confirmed to possess oncogenic activity including its ability to transform NIH/3T3 cells in contrast to wild-type *neu* [3, 4]. The *neuT* gene sequence was found to contain a single point mutation that changed a valine in the transmembrane domain to a glutamic acid (V664E) [5]. In addition to transforming NIH/3T3 cells, *neuT* was able to transform mammary epithelial cells [6]. The transforming potential of the mutated *neu* protein was shown to be due to increased receptor dimerization and kinase activity [7].

 Soon after the discovery of *neuT*, about 30% of human primary breast cancers were found to contain amplification of the *neu *human homolog, *erbB2*/*HER2* [8], located at chromosomal location 17q21 [9]. *HER2 *gene copy number was found to be increased by 2- to over 20-fold in a subset of breast tumors [8]. In contrast to the rat gene, the amplified human *HER2 *gene does not require mutation for oncogenic function; rather overexpression of the wild-type protein can be oncogenic on its own. NIH/3T3 cells transfected with human *HER2* formed colonies in soft agar and tumors in athymic mice [10] when cells expressed amplified levels of *HER2*, demonstrating that amplified and overexpressed human HER2 acts as an oncogene. *HER2 *amplification and overexpression is associated with reduced time to progression and reduced overall survival in breast cancer patients [8]. Although HER2 kinase mutations have been detected in some cancers including lung adenocarcinomas [11], mutation of *HER2 *does not appear to be required to confer transforming potential in mammary epithelial cells, as the amplified *HER2* found in overexpressing tumors is generally wild-type. 

## 3. HER2 Signaling

 Since the initial studies that established *HER2 *amplification in breast cancer, structural and functional analyses have helped to identify the signaling pathways contributing to HER2-mediated cellular transformation. The HER2 monomeric protein has three major regions—the extracellular amino-terminal region comprised of four domains (domains I–IV), the hydrophobic transmembrane domain, and the carboxy-terminal kinase domain comprised of the juxtamembrane domain, tyrosine kinase, and C-terminal tail with autophosphorylation sites. Using a chimeric receptor where the EGFR C-terminus was replaced with the C-terminus of HER2 demonstrated that the HER2 C-terminus can replace the autophosphorylation tyrosine residues in EGFR [12]. Thus, it was concluded that HER2 autophosphorylation sites are located in the C-terminus and are identical to the C-terminal phosphorylation sites of EGFR [12]. *In vitro* kinase phosphorylation of HER2 followed by tryptic digestion and HPLC separation of the phosphopeptides identified the specific phosphorylation sites as Y1248, Y1023, Y1112, Y1127, Y1139, Y1196, Y1221, and Y1222, all within the C-terminus [13]. Although a specific ligand has not been identified for HER2, ligand stimulation of other erbB family members induces receptor heterodimerization with HER2 [14]. HER2 kinase function is not required for homodimerization, as mutation of Lys732 in the ATP-binding site of HER2 results in ablation of the kinase function, and this kinase-dead HER2 can still interact with HER2, as assessed by transfection and coimmunoprecipitation experiments [15]. In contrast, mutation of residues 996–998 in the C-terminal tail of HER2 abrogated the ability of HER2 to form homodimers [15], indicating that these residues are important for receptor-receptor interactions. In addition, mutation of residues 996–998 impaired autophosphorylation of HER2 even when cells were stimulated with heregulin [15]. However, upon stimulation with heregulin, mutant 996–998 HER2 was still able to heterodimerize with HER3, although to a lesser degree than wild-type HER2, and retained the ability to phosphorylate HER3, as HER2 kinase function was still intact [15]. The extracellular domain of HER2 appears to be sufficient for dimerization, as mutant HER2 constructs lacking the intracellular portion of HER2 were still able to interact with full-length EGFR and HER2 [16–18]. Domains I, II, and IV of the extracellular region were involved in HER2-EGFR heterodimerization [19]. The intracellular portion still plays a critical role in mediating optimal receptor-receptor interactions; the relative contributions of various regions of the HER2 protein in facilitating dimerization are comprehensively reviewed elsewhere [20].

 In HER2-overexpressing cells, excess levels of up to 100 times the normal HER2 expression level can result in spontaneous and constitutive ligand-independent dimerization with subsequent activation of the cytoplasmic kinase region [21–23]. Kinase signaling can then stimulate autophosphorylation and downstream signaling, primarily through the PI3K-Akt-mTOR and Ras-Raf-MEK-Erk1/2 pathways. Activation of these pathways promotes proliferation, evasion of apoptosis, angiogenesis, and invasion, leading to tumor growth and progression. Ultimately, which signaling pathway is activated may be a result of whether HER2 is found primarily in homodimers or heterodimers. One study suggested that HER2 homodimers selectively activated Ras-MAPK, while heterodimers activated both MAPK and PI3K signaling pathways [24]. 

 PI3K-mTOR signaling is a major effector of HER2 activity, as PI3K blockade suppressed tumor growth in multiple models of HER2-overexpressing breast cancer [25, 26]. Class Ia PI3Ks are heterodimers of a p85 regulatory subunit and p110 catalytic subunit [27]. Upon growth factor stimulation, p85 binds to membrane bound receptors or adaptor proteins, thus recruiting PI3K to the cell membrane. The p110 catalytic portion of PI3K is then activated by membrane receptors, and phosphorylates PIP2 to PIP3, activating kinase events downstream. The lipid phosphatase PTEN dephosphorylates PIP3 to create PIP2, counteracting the kinase function of PI3K. Three human p110 isoforms exist (alpha, beta, and delta) and are encoded by distinct genes. The p110 alpha catalytic subunit was recently shown to be required for Neu-dependent mammary tumorigenesis in mice [27], arguing for selective inhibition of this PI3K p110 isoform as a potential treatment strategy for HER2-positive breast cancers. 

 In addition to PI3K signaling, MAPK signaling appears to contribute to progression of HER2-positive breast cancer. Stable transfection of HER2 into MCF7 estrogen receptor- (ER-) positive cells (MCF7/HER2) caused hyperactivation of MAPK signaling and resistance to the ER modulator tamoxifen [28]. Combined inhibition of HER2 and MEK using AG1478 and either U0126 or dominant negative MEK1/2, respectively, increased tamoxifen sensitivity. Cotreatment with HER2 kinase inhibitor and tamoxifen suppressed MAPK signaling in association with reduced MCF7/HER2 xenograft tumor growth [28]. Strategies that combine inhibition of PI3K and MEK may achieve the most benefit against HER2-overexpressing cancers, particularly those that are refractory to HER2-targeted treatments. Combined administration of PI3K/mTOR inhibitor BEZ235 and MEK inhibitor AZD6244 to MMTV-*neu* mice achieved nearly complete tumor regression [29]. Similar effects were observed when mice were treated with single-agent lapatinib. As reviewed below, progression on lapatinib is often associated with heightened mTOR or MEK signaling; thus, coinhibition of PI3K/mTOR and MEK is a plausible strategy for treating lapatinib-naïve or lapatinib-refractory HER2-positive breast cancer. 

## 4. Trastuzumab: Mechanisms of Action

 Because it was recognized that constitutive HER2 signaling can drive breast tumor development and progression, strategies were developed to therapeutically target HER2 activity. The first successful approach began with the development of a mouse monoclonal antibody (mAb) [30] called 4D5, which targets an extracellular epitope of the HER2 protein [31]. HER2-specific monoclonal antibody 4D5 blocked phosphorylation of HER2 and suppressed growth of HER2-positive breast cancer cell lines and xenografts [31–34]. In order to translate 4D5 clinically, a derivative that includes the antigen binding loops of 4D5 and the human variable region and immunoglobulin G1 constant domains was constructed [35]. The recombinant humanized anti-HER2 antibody that resulted from this effort is called trastuzumab (Herceptin; Genentech, San Francisco, CA, USA).

 Trastuzumab achieved significant regression of human HER2-overexpressing tumor xenografts as monotherapy or in combination with cytotoxic chemotherapeutic agents [36–38]. The mechanisms underlying the anticancer activity of trastuzumab are not completely known, but several have been proposed (Table 1) [39–44]. Some studies have suggested that trastuzumab downregulates total levels of HER2 on the cell surface [45, 46], which may ultimately reduce downstream PI3K and MAPK signaling. Trastuzumab has been shown to block cleavage of the extracellular domain of HER2, thus, preventing formation of the constitutively active membrane-bound 95-kiloDalton HER2 protein called p95 HER2 [46, 47]. Trastuzumab may also selectively inhibit HER2-HER3 heterodimerization [46, 48], reducing PI3K signaling. Induction of cell cycle arrest by trastuzumab is mediated by p27kip1 and inhibition of cdk2 activity [49, 50]. Furthermore, trastuzumab appears to reduce angiogenesis, which may result in increased permeability of blood vessels [51], potentially increasing delivery of drugs to the tumor. Finally, HER2-positive cells that become “coated” in trastuzumab will be recognized by specific immune cells, causing antibody-dependent cellular cytotoxicity (ADCC), or lysis of the antibody-bound cells [52, 53]. This last mechanism may be quite important to the overall activity of trastuzumab, as trastuzumab was unable to achieve an antitumor response in mice that lacked Fc gamma receptors present on the ADCC-promoting immune cells, natural killer cells and macrophages, and is discussed in detail below as a mechanism of trastuzumab resistance [53]. 

 Trastuzumab was the first HER2-targeted treatment to be approved by the Food and Drug Administration (F.D.A.), with specific approval given in 1998 for treatment of metastatic HER2-overexpressing breast cancer, and in 2006 as adjuvant therapy in combination with chemotherapy for node-positive, HER2-overexpressing breast cancer. Two NCI cooperative group trials, NSABP B-31 and NCCTG N9831, showed that amongst 3752 women with HER2-overexpressing breast cancer who received adjuvant chemotherapy, and radio- or endocrine therapy as appropriate, those who also received trastuzumab were significantly less likely to have disease progression. In the trastuzumab-treated groups, the risk of recurrence was reduced by 52%. In one initial trial of adjuvant trastuzumab in 42 patients with early stage invasive operable HER2-positive breast cancer, 23 received chemotherapy plus trastuzumab and 19 received chemotherapy alone [54]. Twenty of 23 patients receiving trastuzumab (86.9%) showed clinical complete response, compared to 9 of 19 (47.4%) patients in the chemotherapy alone group. The pathologic complete response rate for the trastuzumab group was 65%, compared to 26% in the chemotherapy alone group. The 4-year followup of NSABP B-31 and NCCTG N9831 trials showed sustained significant reduction in disease-free survival for patients treated with trastuzumab [55]. In addition, trastuzumab treatment resulted in 39% reduced death rate, showing significantly improved overall survival benefit when early stage invasive HER2-positive breast cancer is treated with trastuzumab plus chemotherapy. Similarly, favorable response rates have been achieved in patients with metastatic disease when trastuzumab was given as a single agent [56–58] or in combination with chemotherapeutic agents [59–61]. 

 A subset of patients with metastatic disease showed initial resistance to single-agent trastuzumab, although combination with chemotherapy leads to improved response rates. In addition, a subset of patients with early-stage or metastatic disease may eventually acquire resistance to trastuzumab, as is the case with many molecularly targeted cancer therapies. During the past decade, a major focus of research in the field of HER2-overexpressing breast cancer has been the identification of mechanisms of trastuzumab resistance. As a result, more than 600 articles addressing this issue now appear on PubMed. Multiple signaling molecules have been proposed as mediators of resistance and potential new molecular targets for therapeutic intervention in this subtype of breast cancer. In addition, new HER2-specific therapies have been developed and approved for treating metastatic HER2-positive breast cancer. 

 Combining HER2-targeted therapies or combining therapies targeted against mediators of resistance with trastuzumab has shown success in preclinical models of trastuzumab-naïve and resistant breast cancer. In particular, dual inhibition of HER2 achieved by combining trastuzumab with tyrosine kinase inhibitor (TKI) lapatinib or HER2 mAb pertuzumab offers clinical benefit to many patients with HER2-overexpressing metastatic breast cancer. The clinical support for combining HER2-targeted agents and the potential molecular mechanisms of synergy between these agents are discussed below. In addition, we review rationale and strategies for combined blockade of critical pathways mediating resistance and trastuzumab. Finally, we will examine the key issues that must be addressed in order to move the field of trastuzumab resistance forward. 

## 5. Trastuzumab: Potential Mechanisms of Resistance

 We and others have reviewed the literature examining potential mechanisms of resistance to trastuzumab [39, 62, 63]. The major model systems studied to identify mechanisms of resistance have been cell culture based. These include *HER2*-amplified breast cancer cell lines that display primary resistance, meaning they do not undergo growth inhibition upon trastuzumab treatment despite lack of prior trastuzumab exposure. In addition, *HER2*-amplified breast cancer cell lines that demonstrate growth inhibition, that is, sensitivity to trastuzumab, such as SKBR3 and BT474, have been chronically exposed to clinically relevant doses of trastuzumab, allowing for clones with acquired resistance to emerge. In many models of acquired resistance, *HER2* amplification and/or overexpression have been maintained at the parental level, as confirmed by fluorescent *in situ* hybridization (FISH) and Western blotting [64]. A limitation of these models of acquired resistance is lack of *in vivo* selection for resistance; thus, the contributions of the natural tumor environment, tumor-stroma interactions, and host factors were ignored in development of these models. Future studies can improve upon these models by treating orthotopic xenografts of trastuzumab-sensitive cells until acquired resistant tumors emerge, and then isolating these tumors or their counterparts which retain sensitivity, and culturing these *ex vivo*. Similarly, HER2 transgenic mice may be allowed to form mammary tumors and then treated with trastuzumab until resistant tumors emerge, and these can be cultured for further analysis. Finally, *ex vivo* analysis of patient tumors will add significantly to the literature. Tumors can be isolated and cultured to create primary models or implanted in mice to create xenografts. In addition to improved models, the endpoint for experimental therapeutic studies in models of trastuzumab resistance should not be restricted to growth inhibition or apoptosis, but should include analysis of metastatic burden *in vivo* or invasion and epithelial-to-mesenchymal (EMT) *in vitro*. Using currently available models, potential mechanisms of trastuzumab resistance that have been identified include epitope masking, truncated HER2, cross-signaling or compensatory signaling by other cell surface receptors, altered downstream signaling, and impaired immune function. 

### 5.1. Epitope Masking: Mechanism of Trastuzumab Resistance

Membrane mucins such as Muc4 have been shown to reduce matrix-cell interactions and adhesion [65] and to stimulate HER2 signaling via an EGF-like domain [66]. Overexpression of Muc4 has been reported in some aggressive mammary tumors [67, 68]. Stable transfection of Muc4 reduced binding of 1 *μ*g/mL trastuzumab to HER2 by 50% without any change in total HER2 expression, although higher concentrations of trastuzumab (10 or 100 *μ*g/mL) were still able to bind [67]. In MCF-7 cells, which express wild-type (nonamplified) levels of HER2, overexpression of Muc4 blocked binding of trastuzumab to HER2 at all tested concentrations of trastuzumab (1, 10, or 100 *μ*g/mL) [67]. Coimmunoprecipitation experiments showed that Muc4 and HER2 interact [67]. A subsequent report [69] showed increased expression of Muc4 in JIMT-1 HER2-overexpressing breast cancer cells which display primary resistance to trastuzumab versus sensitive cell lines. Knockdown of Muc4 in JIMT-1 cells increased trastuzumab binding [69]. Interestingly, matrix metalloproteinase activation by APMA caused cleavage of membrane proteins including Muc4 in JIMT-1, leading to increased binding of trastuzumab [69], suggesting that Muc4 interaction with HER2 results in epitope masking such that trastuzumab cannot recognize and bind to HER2. More recently, upregulation of mucins including Muc4 was reported in ER-positive/HER2-positive breast cancer xenografts that had acquired resistance to trastuzumab plus lapatinib [70]. 

### 5.2. Truncated HER2: Mechanism of Trastuzumab Resistance

Trastuzumab has been shown to block cleavage of HER2 ECD and prevent formation of constitutively active p95HER2 [47]. However, in cancers that already express p95HER2, trastuzumab is unlikely to inhibit kinase activity of the truncated receptor which lacks the ECD binding epitope. Phosphorylation and dimerization of p95HER2 has been shown to be stimulated by the HER3 ligand heregulin, causing interaction with HER3 but not EGFR [71]. Trastuzumab could not block p95HER2 signaling; in contrast, the EGFR/HER2 kinase inhibitor lapatinib suppressed HER2 signaling through PI3K and MAPK [71, 72]. In one study, 9 of 46 patients with HER2-positive disease expressed p95HER2; only one of the nine showed a response to trastuzumab [72]. In contrast, 19 of 37 patients expressing full-length HER2 achieved response to trastuzumab [72]. Additional studies have confirmed lapatinib efficacy in the setting of p95HER2-positive disease in contrast to trastuzumab [73–75]. Truncated isoforms of HER2 may result from MMP-based cleavage or alternative translation start sites [76, 77] and are reviewed elsewhere [78]. P95HER2 (648CTF) is formed by MMP cleavage of full-length HER2 and remains bound at the membrane [47]. There is also a p95HER2 that is present in the cytoplasm, and additional forms expressed in cell nuclei have been described [79]. The truncated isoform p110 (611CTF) arises from alternative translation [80] and is associated with increased migration, invasion, and transformation of normal human mammary epithelial cells, causing *in vivo* tumor formation [79]. These studies demonstrate that oncogenic HER2 exists in multiple isoforms, and that therapeutic approaches must be based upon expression of individual isoforms. Antibody-based approaches may retain efficacy in the context of overexpressed full-length HER2, whereas kinase inhibition (i.e., lapatinib) may be most effective at suppressing progression of tumors that express truncated HER2 isoforms.

### 5.3. Cross-Signaling to HER2: Mechanism of Trastuzumab Resistance

 In addition to reduced drug-target interactions possibly due to epitope masking, sustained activation of HER2 in the presence of trastuzumab treatment has been reported. Several signaling molecules appear to activate cross-talk to HER2, including insulin-like growth factor-I receptor (IGF-IR), hepatocyte growth factor (HGF) and its receptor Met, growth differentiation factor 15 (GDF15), and members of the erbB family. We have recently reviewed the literature supporting IGF-IR cross-talk and/or overexpression in the development of trastuzumab resistance [81]. Lu et al. [82] first demonstrated that increased IGF-IR signaling due to stable overexpression of IGF-IR in SKBR3 cells can abrogate trastuzumab-mediated G1 arrest. IGF-IR signaling was inhibited using IGF-binding protein (IGFBP) 3, which restored the growth inhibitory activity of trastuzumab [82, 83]. We and others [81, 84–86] have shown that IGF-IR and HER2 can interact, facilitating cross-talk such that IGF-I induces phosphorylation of HER2, and IGF-IR inhibition reduces HER2 phosphorylation. IGF-IR/HER2 interactions have been reported in both models of acquired trastuzumab resistance [81, 85, 86], as well as in BT474 and MCF7/HER2 stable transfectant, which are sensitive to trastuzumab [84]. The exact mechanisms by which IGF-IR promotes trastuzumab resistance, the mechanisms mediating interactions between these receptors, and the mechanisms underlying growth suppressive effects of IGF-IR-targeted therapies [86, 87] are unclear and warrant further study. 

 The Met receptor tyrosine kinase was found to be highly expressed in 5 of 20 examined HER2-overexpressing breast cancer tissues, although a statistically significant correlation between HER2 and Met expression was not present [88]. The Met ligand HGF can promote invasiveness of HER2-expressing breast cancer cells through a MEK-dependent mechanism that results in reduced E-cadherin and internalization of ZO-1, indicative of basement membrane breakdown and EMT [89]. Costimulation of Met and HER2 with HGF and neuregulin-1 beta, respectively, resulted in increased phosphorylation of downstream Akt and Erk1/2 and increased proliferation, as measured by MTT assay [90]. Coinhibition using Met siRNA or Met kinase inhibitor SU11274 plus trastuzumab resulted in greater inhibition of proliferation versus either approach alone and was associated with greater inhibition of Akt phosphorylation [90]. Recently, a group of HER2-positive metastatic breast cancer tissues was examined by FISH for *Met *(*n* = 130) and *HGF* gene copy number (*n* = 84) [91]. *Met* amplification (FISH-positivity) was significantly associated with resistance to trastuzumab (44.4% versus 16% in nonamplified) and shorter time to progression (5.7 versus 9.9 months). *HGF* amplification was also significantly associated with resistance (30.3% versus 7.8%), providing clinical evidence that Met/HGF overexpression is associated with trastuzumab resistance. These studies provide rationale for current trials testing Met inhibition against HER2-positive breast cancer that has progressed on trastuzumab. These trials include dual blockade of Met and vascular endothelial growth factor receptor (VEGFR) in trastuzumab-refractory populations. The role of VEGFR signaling in trastuzumab resistance has been reviewed elsewhere [39].

 Another more recently described mechanism of trastuzumab resistance is elevated expression of GDF15 [92]. GDF15 is found at chromosomal location 19p13.11 and encodes a disulfide-linked homodimer-secreted cytokine of approximately 34 kiloDaltons, structurally similar to the TGF beta cytokine [93]. Increased circulating levels of GDF15 have been reported in patients with breast, prostate, ovarian, and pancreatic cancers [94–98], including elevated serum levels of GDF15 reported in 6 of 10 (60%) metastatic breast carcinomas [98]. We [92] showed by gene microarray, real-time PCR, and ELISA assays that the transcript and endogenous and secreted GDF15 protein are overexpressed in cells with acquired or intrinsic trastuzumab resistance. Stimulation with exogenously added recombinant human GDF15 or stable transfection of a GDF15 expression plasmid reduced trastuzumab sensitivity. The receptor for GDF15 has not been identified; however, a potential cross-talk is activated by GDF15, which induced phosphorylation of HER2 and downstream Akt and Erk1/2 in HER2-overexpressing SKBR3 [99, 100] and BT474 [92] breast cancer cells. The HER2 tyrosine kinase inhibitors (TKI) lapatinib and AG879 blocked GDF15-mediated HER2 signaling and reduced GDF15-mediated trastuzumab resistance [92]. Stimulation of HER2-overexpressing cells with GDF15 also induced phosphorylation of TGF beta receptor substrate Smad2 and nonreceptor tyrosine kinase Src, both of which were blocked after treatment with the TGF beta receptor type II kinase inhibitor SB431542. In contrast, HER2 kinase inhibition did not block GDF15-mediated Src phosphorylation [92], suggesting that TGF beta receptor signaling, and not HER2 signaling, mediates Src phosphorylation and may contribute to GDF15 cross-talk to HER2.

 HER2 interacts with erbB family members EGFR and HER3. Increased levels of EGFR and HER3 ligands have been associated with reduced response to trastuzumab and have previously been reviewed in this context [39]. In addition, coexpression of EGFR and/or HER3 is frequently found in HER2-positive disease. Amongst 155 patients treated with trastuzumab, 15% showed overexpression of EGFR [101]. EGFR expression and vascular invasion were significantly associated with overall survival of patients with metastatic disease. The same study [101] showed that IGF-IR overexpression and inactivating phosphorylation of the proapoptotic protein Bad were correlated with resistance to adjuvant trastuzumab. 

### 5.4. Hyperactivated Downstream Signaling: Mechanism of Trastuzumab Resistance

 Activation of downstream signaling pathways can also override growth inhibition by trastuzumab. These mechanisms have been reviewed elsewhere [39] and include increased signaling from PI3K, focal adhesion kinase (FAK), and Src. Constitutive activation of PI3K occurs because of hyperactivating *PIK3CA *mutations [102], reduced PTEN expression [102, 103], or deregulated signaling from upstream growth factor receptors. As an example of oncogene addiction, *HER2* amplification is generally responsive to HER2 kinase inhibition which suppresses downstream PI3K and MEK signaling [104]. Failure to maintain suppression of these downstream pathways results in resistance to HER2-targeted therapies. HER2-overexpressing breast cancers that have progressed on trastuzumab show particular sensitivity to PI3K inhibition [25, 105], indicating that this downstream signaling pathway plays a critical role in driving progression of HER2-postive disease. In contrast to lung cancers that are addicted to EGFR signaling, PI3K inhibition alone can induce apoptosis of HER2-addicted breast cancers [104, 106]. Multiple proapoptotic proteins are regulated by PI3K signaling including survivin and BIM [107]. Incomplete inhibition of PI3K signaling may prevent induction of these proteins, preventing apoptosis in response to HER2-targeted treatments such as lapatinib [107]. 

### 5.5. Failure to Activate ADCC: Mechanism of Trastuzumab Resistance

 Impaired stimulation of an ADCC response has been associated with trastuzumab resistance. Immune cells including natural killer (NK) cells, monocytes, and macrophages express Fc gamma receptors such as CD16 [53]. When antibodies bind to the cell surface of host cells, such as trastuzumab binds to HER2 on breast cancer cells, Fc gamma receptors of immune cells recognize the Fc portion of antibodies. These immune cells then release cytokines and cytotoxic granules enter the target cell, inducing apoptosis. Mice that lacked activating Fc receptors or in which interaction between antibody and NK cells had been disrupted showed less effective ADCC lysis of HER2-positive tumor cells upon treatment with trastuzumab [53]. Thus, Fc receptor interactions and ADCC contribute to response to trastuzumab. In addition, complete or partial remission of patients treated with neoadjuvant trastuzumab was correlated with tumor infiltration of immune cells and higher *in vitro* ADCC activity in lysis assays [108]. IHC analysis demonstrated a higher number of NK cells and lymphocyte-associated cytotoxic granules in tumors from patients with HER2-positive breast cancer treated with trastuzumab plus docetaxel [52]. Although there was a trend correlating immune cell infiltration with responsiveness, this did not reach statistical significance in this pilot study [52]. In HER2-positive operable breast cancer, ADCC was achieved by trastuzumab in 15 of 18 patients [109]. One patient who had a pathologic complete response showed the most intense ADCC, whereas four others who had partial responses showed intermediate ADCC. Lack of response to trastuzumab was associated with inability to mount an ADCC response [109]. 

 Cytokines that stimulate production of NK cells, such as IL-2, have been shown to promote lysis of 4D5-coated MCF7/HER2 cells, as measured by ^51^Cr-release cytotoxicity assay [110]. Similarly, HER2-positive gastric cancer cells isolated from patients with advanced disease showed reduced NK-mediated ADCC, which was improved *in vitro* upon stimulation with IL-2 [111]. Amongst 10 patients with HER2-overexpressing breast cancer who received trastuzumab plus IL-2, one partial response, 5 cases of stable disease and 4 cases of progressive disease were reported [112]. Although an increased number of NK cells and ADCC were documented, these did not correlate with clinical response. Another study failed to show benefit from the addition of IL-12 to trastuzumab in the majority of the 12 patients with HER2-positive breast cancer [113]. However, one complete response in a HER2-positive patient and 2 cases of stable disease lasting longer than 6 months were documented [113]. Another approach to stimulate ADCC was recently shown by cotreating with trastuzumab plus a CD137 agonistic antibody, which bound and stimulated CD137 on NK cells to promote ADCC [114]. Thus, strategies that stimulate ADCC may improve response to trastuzumab. In addition, combination approaches that are currently being tested against trastuzumab-refractory cancers may enhance ADCC. For example, treatment with a combination of HER2-targeted antibodies that targeted distinct epitopes was shown to induce a greater level of ADCC *in vitro* than when cells were treated with trastuzumab alone [115]. The importance of mounting an ADCC response is also discussed below as a potential mechanism driving synergy between trastuzumab and lapatinib.

## 6. Combining HER2-Targeted Therapies

### 6.1. Lapatinib

Lapatinib (Tykerb; GlaxoSmithKline) is a dual EGFR/HER2 kinase inhibitor that reduces EGFR and HER2 signaling and induces apoptosis in multiple models of HER2-overexpressing breast cancer [116, 117]. Although lapatinib blocks EGFR kinase activity, the contribution of this function to anticancer activity in HER2-positive breast cancer cells appears to be minimal, as loss of HER2 expression reduced lapatinib efficacy, while loss of EGFR expression did not [118]. Lapatinib plus capecitabine is approved for HER2-overexpressing breast cancers that have progressed on trastuzumab (second-line), and as first-line therapy combined with letrozole for ER-positive, HER2-positive metastatic breast cancer. When lapatinib was combined with chemotherapy, an overall response rate of 22% and clinical benefit rate of 27% was achieved with a median time to progression of 8.4 months [119]. Lapatinib was still effective as a single agent in tumors that had received prior trastuzumab exposure, achieving clinical benefit rates ranging from 12.4% to 25% [120, 121]. 

 Response to single-agent lapatinib has been associated with multiple transcription factors and signaling pathways. HER2-positive lines whose proliferation was inhibited by nanoMolar concentrations of lapatinib showed inhibition of Akt/mTORc1 signaling in contrast to nonresponsive cell lines [122, 123]. In addition, *PIK3CA *mutation was associated with reduced induction of apoptosis in response to lapatinib [107], suggesting that the ability to inhibit PI3K signaling is required for optimal response to lapatinib. Inhibition of Akt resulted in an increased expression and phosphorylation of Fox03a transcription factor in responsive cell lines [123]. Interestingly, Fox03a upregulates ER signaling, which has been documented as a potential mechanism of lapatinib resistance [123, 124], perhaps due to inhibition of apoptosis mediated by Bcl-2 family proteins [125]. A role for anti-apoptotic Bcl-2 family members has also been implicated in trastuzumab resistance, with increased Bcl-2 : Bax ratios reported in cells with acquired trastuzumab resistance [126]. 

 In addition to changes in signaling which may reduce sensitivity to lapatinib, recent evidence indicates that *HER2* kinase domain mutations are associated with reduced lapatinib efficacy. Somatic mutations in the HER2 kinase domain have been reported in multiple solid tumor types, including breast, lung, and head and neck [127–129]. Mutations localize to the ATP binding site, the gatekeeper residue, or the activation loop [130]. Mutant HER2 protein retains autophosphorylation, downstream signaling, and heterodimerization abilities [130]. Compared to wild-type HER2, mutants showed enhanced transformation potential and differential sensitivity to lapatinib [130]. Mutation T798M at the gatekeeper residue and an ATP binding site mutation (L755S and L755P) showed reduced response to lapatinib, with IC50s of up to 50 times higher relative to wild-type HER2 [130]. The gatekeeper mutant displayed higher affinity for ATP, providing a potential explanation for resistance to the reversible TKI lapatinib. In contrast, irreversible TKIs CL-387785 and WZ-4002 bound irreversibly to an active conformation of HER2 and suppressed HER2 signaling and growth in stable clones of wild-type or mutant T798M, L755P, or L755S HER2 [130]. Much of the work correlating HER2 mutations with TKI resistance has been done in tumor types other than breast. Additional studies should examine HER2-positive breast tumors for HER2 kinase domain mutations and differential response to HER2-targeted reversible TKI lapatinib versus irreversible inhibitors. 

 Lapatinib appears to retain efficacy in some models of HER2-positive cancer that have progressed on prior trastuzumab treatment [131]. Combined treatment with trastuzumab and lapatinib in trastuzumab-naïve HER2-overexpressing breast cancer cell lines showed synergistic inhibition of proliferation [131]. Further, the combination of trastuzumab plus lapatinib was more effective than lapatinib alone in patients who had received prior trastuzumab treatment [120]. The clinical benefit rate for the combination was initially reported as 24.7% versus 12.4% for lapatinib alone [120]. Although a trend for improved overall survival was observed in the combination arm, this had not yet reached statistical significance at the time that the first study was reported [120]. However, it was noted that amongst trastuzumab-refractory patients with better performance status, few metastatic sites, and no liver metastases, the combination of lapatinib and trastuzumab achieved significantly higher progression-free survival (PFS) versus lapatinib alone [120]. More recently, longer term evaluation of 291 patients showed that median PFS and overall survival both reached statistical significance for the combination arm [132]. Median overall survival was 14 months for those treated with trastuzumab plus lapatinib and 9.5 months for lapatinib treatment alone. Thus, the combination of trastuzumab and lapatinib achieves superior PFS and OS when compared to lapatinib alone amongst patients previously treated with trastuzumab. A meta-analysis [133] of trials that compared either trastuzumab plus neoadjuvant chemotherapy to lapatinib plus neoadjuvant chemotherapy (GeparQuinto [134] and GEICAM2006-14) or combination trastuzumab plus lapatinib with neoadjuvant chemotherapy versus trastuzumab plus neoadjuvant chemotherapy (NeoALTTO [135], CHER-LOB [136], and NSABP B-41) was performed. The absolute pathologic complete response rate (pCR) for those treated with trastuzumab and chemotherapy was 36% versus 29% when treated with lapatinib and chemotherapy [133]. For the trials that tested combination HER2 blockade, the pCR rate was 53% for the combination and chemotherapy versus 39% for trastuzumab and chemotherapy.

 Several molecular mechanisms are likely to account for the benefit achieved by combined treatment with trastuzumab and lapatinib. Due to several nonoverlapping mechanisms of resistance, lapatinib may suppress signaling pathways that mediate resistance to trastuzumab. For example, increased expression [82] or cross-signaling [85, 86] from IGF-IR reduces trastuzumab-mediated growth inhibition. However, lapatinib appears to retain cytotoxic activity in these cells [137]. Another report [138] suggested that increased phosphorylation of IGF-IR, HER3, or EGFR correlates with response to lapatinib, but not to trastuzumab. Another potential mechanism of trastuzumab resistance is mediated by increased expression of growth differentiation factor 15 (GDF15) [92], which has been shown to induce phosphorylation of HER2 [92, 99]. Lapatinib treatment suppressed GDF15-mediated HER2 phosphorylation [92], suggesting that lapatinib may remain effective in cancers that are resistant to trastuzumab due to upregulation of GDF15. Thus, lapatinib may suppress HER2 kinase activity to overcome compensatory signaling mechanisms that cause trastuzumab resistance due to sustained phosphorylation of HER2. In addition, lapatinib has been shown to upregulate HER3. HER3 possesses multiple PI3K binding sites in its cytoplasmic tail; as a result, HER2/HER3 represents the most potent erbB dimer pair due to activation of PI3K signaling [139–141]. Lapatinib-mediated upregulation of HER3 resulted in sustained PI3K signaling [142]. HER3 knockdown or pharmacologic blockade improved response to lapatinib [142]. 

 Lapatinib and trastuzumab appear to differ in their effects on expression and dimerization of erbB/HER family members. In cell line models of trastuzumab-naïve HER2-positive breast cancer (SKBR3 and MCF7/HER2), lapatinib blocked phosphorylation of HER2 and reduced ubiquitination and subsequent degradation of cell surface HER2 [143]. The result was increased levels of total HER2 within 12–24 h in cells treated with lapatinib or within 36–48 h in cells treated with lapatinib plus trastuzumab, while trastuzumab alone resulted in downregulation of HER2 expression within 24 h of treatment [143]. Treatment of HER2-positive cancer cell xenografts with lapatinib or lapatinib plus trastuzumab resulted in increased staining for HER2 at the cell surface [143]. The accumulation of HER2 in cell lines treated with lapatinib corresponded with increased heterodimerization between HER2 and EGFR or HER3 [143]. However, these dimers were inactive, as phosphorylation of HER2 was suppressed in the presence of lapatinib alone or when combined with trastuzumab [143]. 

 In addition to inducing cell surface accumulation of HER2, lapatinib achieved a significant increase in trastuzumab-mediated ADCC activity [143]. A more recent report [144] further confirmed the findings that lapatinib increases expression of cell surface HER2 in the HER2-positive SKBR3 and BT474 lines and enhances trastuzumab-mediated ADCC by almost twofold. Since an impaired ability to mount an effective ADCC response is a potential mechanism of escape from trastuzumab, the ability of lapatinib to restore this effect when combined with trastuzumab is a potential mechanism of synergy between the two HER2-targeted drugs. 

 Structural modeling of the HER2 dimers bound to lapatinib showed that HER2 homodimers and heterodimers are more stable when lapatinib is bound [143]. In fact, time-resolved fluorescence resonance energy transfer (TR-FRET) performed in HER2-overexpressing ovarian cancer-derived cells (SKOv3) showed that trastuzumab, the HER2 mAb pertuzumab, or EGFR mAb cetuximab disrupted EGFR/HER2 heterodimer formation by 44%, 24%, or 48%, respectively, versus 72% heterodimer disruption by combined trastuzumab and cetuximab and little disruption of heterodimer formation by lapatinib [145]. Thus, trastuzumab and lapatinib appear to differ in their effects on erbB dimerization, and combined treatment could potentially increase disruption of dimers, similar to what was observed with combined trastuzumab and EGFR mAb cetuximab. 

### 6.2. Pertuzumab

 The HER2 mAb pertuzumab (2C4, Omnitarg; Genetech) binds to domain II of HER2 [146], whereas trastuzumab binds to domain IV [147]. Domain II is a critical region of receptor-receptor interaction, compared to domain IV, which does not appear to directly regulate dimerization. As a result of binding to domain II, pertuzumab sterically hinders interactions of HER2 with other receptors. Disruption of baseline as well as ligand-stimulated HER2-HER3 and HER2-EGFR dimerization has been documented upon pertuzumab treatment of HER2-positive SKBR3 breast cancer cells or MCF7 breast cancer cells, which express wild-type (nonamplified) levels of HER2 [148]. Further, FRET analysis indicated that trastuzumab increased formation of HER2 homodimers, while pertuzumab reduced homodimerization [149]. Disruption of dimerization by pertuzumab has been associated with reduced HER2 phosphorylation and growth inhibition of BT474 and MCF7 xenograft tumors [148]. 

 A phase I trial tested trastuzumab plus pertuzumab in a trastuzumab-refractory population of 11 patients and showed a favorable response rate of 18% with median time to progression of 6 months [150]. A subsequent phase II trial similarly showed a 22.4% response rate amongst 66 patients with a clinical benefit rate of 50%; median progression-free survival was 5.5 months [151]. The CLEOPATRA trial including 808 patients treated with pertuzumab, trastuzumab, and docetaxel versus trastuzumab plus docetaxel as first-line treatment against HER2-positive metastatic breast cancer showed significant improvement in median progression-free survival of 18.5 versus 12.4 months, respectively [152]. Further, as neoadjuvant therapy, the pertuzumab-trastuzumab-docetaxel combination achieved higher pathologic complete response (45.8% versus 29% in the trastuzumab-docetaxel arm) [153]. 

 We previously showed that combining trastuzumab and pertuzumab in HER2-positive BT474 cells resulted in synergistic inhibition of proliferation, increased apoptosis, disruption of HER2-HER3 dimerization, and reduced Akt phosphorylation [154]. Xenograft models of BT474 or MCF7/HER2 showed increased median time to tumor progression (49 days) when treated with combination trastuzumab, pertuzumab, and EGFR TKI gefitinib versus pertuzumab (28 days) [155]. Inhibition of HER2 signaling and proliferation and induction of apoptosis were enhanced upon treatment with the combination versus pertuzumab [155]. In addition, BT474 tumors showed complete regression when treated with trastuzumab, pertuzumab and gefitinib and remained absent for up to almost 8 months [155]. HER2-overexpressing KPL-4 breast xenografts and Calu-3 nonsmall cell lung cancer cell xenografts, including those that had progressed on prior trastuzumab treatment, showed significant regression when combination trastuzumab plus pertuzumab was administered [156]. In contrast to the combination of lapatinib and trastuzumab, addition of pertuzumab does not appear to increase ADCC; rather both antibodies induce ADCC on their own, without any further increase when given together [156]. However, the antibody combination promoted cell cycle exit and apoptosis. Under low-serum cell culture conditions, trastuzumab induced quiescence in 38% of BT474 cells [149, 157]. Cotreatment with pertuzumab further increased the percentage of cells entering quiescence to 49%. The combination of antibodies also reduced proliferation, as measured by flow cytometry and Ki-67 staining, more significantly than either antibody alone in BT474 cells [157]. In contrast, apoptosis was induced in only a small percentage of cells, with a significant increase in apoptosis seen when trastuzumab and pertuzumab were combined [157]. 

 The growth suppressive activity of pertuzumab appears to be related to disruption of HER2-HER3 dimerization, resulting in reduced PI3K signaling. In fact, HER3 is now believed to contribute significantly to the growth of HER2-overexpressing breast cancers. In a panel of six *HER2*-amplified breast cancer lines, HER3 siRNA was as effective as HER2 siRNA at inhibiting proliferation of four lines (BT474, HCC1419, SKBR3, and ZR75-30), and more effective than HER2 siRNA in one of the lines (HCC1954) [158]. The antiproliferative effect of HER3 siRNA was associated with HER3 knockdown and reduced phosphorylation of Y1289-HER3 without any off-target effect on HER2. In contrast, EGFR siRNA had no effect on proliferation of any of the six cell lines. Further, inducible HER3 shRNA expression in a BT474-derived line resulted in a 3-fold reduction of 3-d culture growth, and dramatic suppression of xenograft tumor growth with reduced Ki-67 and p-S6 staining and reduced p-Akt levels [158]. Bispecific HER2/HER3 antibodies are being tested clinically against HER2-positive breast cancers. MM-111, a bispecific HER2/HER3 antibody, reduced HER2/3-PI3K signaling, suppressed HER2-positive xenograft tumor growth, and showed synergy with trastuzumab or lapatinib against HER2-positive breast cancers *in vivo* [159].

 Similar to HER3 shRNA and antibodies that target both HER2 and HER3, HER3 surrobodies suppressed PI3K signaling, proliferation, and xenograft tumor growth of HER2-positive cells [160]. Of potential translational importance, the HER3 surrobodies showed stronger antitumor activity combined with trastuzumab versus pertuzumab and trastuzumab combined and were as effective in combination with lapatinib as was trastuzumab plus lapatinib against HER2-overexpressing breast xenografts. Thus, HER3 contributed to tumor growth of HER2-overexpressing breast cancers, suggesting that HER2 oncogenic activity may be due in part to HER3 and its interaction with the PI3K signaling pathway.

 Trastuzumab-resistant cells retained sensitivity to pertuzumab and showed evidence of partial disruption of IGF-IR/HER2 dimerization in resistant cells [86]. Since pertuzumab binds to the dimerization domain (domain II) of HER2, interactions and cross-signaling between HER2 and non-erbB receptors may be disrupted, in addition to disrupting interactions with erbB family members. Thus, cross-activation mechanisms that sustain trastuzumab resistance may be overcome by cotreatment with pertuzumab. In addition, trastuzumab has been shown to block cleavage of the extracellular domain (ECD) of HER2, suppressing formation of constitutively active p95HER2 [47]. In contrast, pertuzumab does not appear to suppress p95HER2 formation, but does disrupt HER2 dimerization, a mechanism distinct from trastuzumab. The presence of non-overlapping mechanisms of action produces significant synergy between two HER2 monoclonal antibodies that bind to different epitopes of the HER2 extracellular region. 

## 7. Future Directions

### 7.1. Translating the Role of IGF-IR in Trastuzumab Resistance Into the Clinic

 We have recently reviewed the literature implicating IGF-IR overexpression and cross-talk in trastuzumab resistance [81]. Targeting IGF-IR in treatment of cancer has lost some enthusiasm over the past few years, as trials have failed to show benefit. However, additional investigations that incorporate biomarker-based patient selection and more complete inhibition of IGF-IR and IGF-IR/insR signaling may be warranted and have recently been reviewed [161]. Blockade of IGF-IR using mAbs does not appear to block insulin receptor signaling, which is advantageous for maintaining glucose homeostasis, but may also result in incomplete suppression of IGF-IR/insulin receptor hybrid signaling, potentially contributing to the observed lack of clinical benefit. Further, the lack of biomarker-based selection of patients in these trials may have obscured the subset of patients that derived benefit, if any. IGF-IR inhibition should be tested in a selected group of patients with trastuzumab-refractory disease based on biomarkers indicative of IGF-IR signaling activation. Analysis of treatment sequencing may also be warranted, as one study demonstrated that chemotherapy followed by IGF-IR inhibition blocked growth of breast cancer cells, whereas inhibiting IGF-IR first actually suppressed chemotherapy-induced apoptosis [162]. Finally, there is a need to better understand the molecular mechanisms by which IGF-IR promotes cancer cell growth, EMT, motility, invasion, suppression of apoptosis, and therapeutic resistance. Multiple signaling pathways may cooperate simultaneously with IGF-IR to drive trastuzumab resistance, such as increased FAK, GDF15, or VEGF signaling, or reduced immune stimulation of ADCC. Ultimately, multiple strategies may have to be combined to cotarget mechanisms of resistance, rather than blocking one upstream molecule such as IGF-IR. For example, combining trastuzumab, pertuzumab, and IGF-IR inhibition may result in greater induction of ADCC, apoptosis, and suppression of invasion. Further preclinical studies are required to determine how IGF-IR inhibition fits into treatment of HER2-overexpressing breast cancer. 

 IGF-IR activation may serve as a biomarker of resistance to trastuzumab. IGF-IR overexpression was associated with worse progression-free survival in patients treated with adjuvant trastuzumab [101], and poor response to preoperative trastuzumab plus chemotherapy [163]. A multivariate analysis also showed that high IGF-IR expression was associated with worse prognosis specifically amongst HER2-positive patients [164]. Studies should incorporate analysis of not only total IGF-IR expression levels, but also markers of IGF-I signaling activation including phosphorylated IRS-1/2 and expression of IGFBPs, and correlate these with response to trastuzumab. 

### 7.2. Understanding the Role of the Estrogen Receptor in HER2-Dependent Breast Cancer

There is now strong evidence of differential response to trastuzumab in ER-positive versus ER-negative HER2-overexpressing breast cancer. Increased HER2 signaling has been well documented in models of acquired resistance to ER-targeted therapies, with current efforts focused on testing anti-HER2 strategies against tamoxifen-resistant disease [28, 165, 166]. In contrast, fewer studies have focused on the role of ER signaling in resistance to HER2-targeted therapies. Xia et al. [124] first demonstrated that acquired resistance to lapatinib was associated with increased ER signaling in preclinical models. Cell line models of HER2-positive breast cancer that had acquired resistance to lapatinib or to lapatinib plus trastuzumab showed increased expression of ER or a downstream target of ER (progesterone receptor, IGF-IR, Cav-1) [125]. In contrast, trastuzumab-resistant lines showed reactivation of erbB signaling, but not increased ER signaling [125]. We previously reviewed the data suggesting that the pathologic complete response rates to trastuzumab-based treatment are lower in patients with the highest ER expression levels in HER2-positive breast cancer [167]. In-depth analysis of a larger population of HER2-overexpressing metastatic breast cancer patients is warranted to determine if indeed reduced response to trastuzumab is associated with higher ER expression and/or signaling. A potential treatment strategy is to simultaneously block ER and HER2, avoiding chemotherapy, in ER-positive/HER2-positive cancers. Again, molecular profiling will be critical for identifying those individuals who will benefit from such an approach versus patients who should continue to be treated with trastuzumab plus chemotherapy or another combination of HER2-targeted therapies.

### 7.3. Establishing Biomarkers

 Multiple therapeutic approaches are now available for treating HER2-positive breast cancer. Molecular profiling of individual tumors may allow for rational selection of the optimal therapeutic strategy. Examples of biomarkers have been discussed above and include truncated HER2 isoforms, PI3K activation, and IGF-IR. Cell cycle regulators including upregulation of Bcl-2 [126] and survivin, downregulation of Fox03a, and reduced expression of p27kip1 [64] may serve as molecular predictors of resistance to trastuzumab. Serum biomarkers including HER2 ECD, erbB ligands, and GDF15 may also be useful at predicting trastuzumab resistance. Serum markers offer a noninvasive method for predicting probability of responding to trastuzumab. Ultimately, creating a molecular signature of trastuzumab response or trastuzumab resistance may allow the best HER2-targeted therapy or combination of therapies to be selected.

## 8. Conclusions

The development of trastuzumab represents a major advance in the treatment of breast cancer. Subsets of patients who acquire resistance to trastuzumab now have multiple therapeutic regimens available to further prolong progression-free survival. Molecular insights into resistance have illustrated bench-to-bedside science at its best, creating new combinations of trastuzumab with inhibitors of signaling pathways that mediate resistance. Future studies will have to address how to best select the optimal therapeutic strategy for each patient. Molecular profiling using cutting-edge genomic and proteomic approaches will allow for individualized approaches to be implemented and will help to establish a molecular signature predicting trastuzumab resistance. These molecular studies will allow patients to receive the most appropriate HER2-targeted treatment combination for their specific tumor profile, ultimately improving overall survival for patients with metastatic HER2-overexpressing breast cancer.

## Figures and Tables

**Table 1:** Proposed mechanisms of action of HER2-targeted therapies.

Drug	Trastuzumab	Lapatinib	Pertuzumab
Effects on HER2 expression	Potential down-regulation of HER2 from the cell surface; blocks cleavage of HER2 ECD, suppressing formation of p95HER2	Accumulation/increased expression of total HER2 at the cell surface	
Effects on dimerization	Potentially partial disruption of dimerization		Sterically hinders interactions of HER2 with other receptors
Effects on downstream signaling	Inhibits PI3K and MAPK signaling	Inhibits PI3K and MAPK signaling	Inhibits PI3K and MAPK signaling
Immune effects	Stimulates ADCC		Stimulates ADCC
Effects on cell cycle regulators	Induction of p27, p21; cdk2 inhibition	Induction of p27; cdk2 inhibition	Induction of p27, p21; cdk2 inhibition
Effects on apoptotic regulators	Down-regulation of Bcl-2, surviving, and Mcl-1	Down-regulation of survivin and Mcl-1; up-regulation of BIM and Fox03a	Induces apoptosis, although mechanisms are unknown
